# Thiazide diuretic-caused hyponatremia in the elderly hypertensive: will a bottle of Nepro a day keep hyponatremia and the doctor away? Study protocol for a proof-of-concept feasibility trial

**DOI:** 10.1186/s40814-018-0263-y

**Published:** 2018-04-06

**Authors:** Marcel Ruzicka, Brendan McCormick, Peter Magner, Tim Ramsay, Cedric Edwards, Ann Bugeja, Swapnil Hiremath

**Affiliations:** 1 Division of Nephrology, Department of Medicine, The Ottawa Hospital, 1967 Riverside Drive, Ottawa, K1H 7W9 Canada; 2 Division of Cardiology, University of Ottawa Heart Institute, Ottawa, Canada; 3 Centre for Practice Changing Research, Ottawa Hospital Research Institute, Ottawa, Canada

**Keywords:** Hyponatremia, Clinical trial, Thiazide, Protein supplementation

## Abstract

**Background:**

Hypertension is the most common modifiable risk factor for cardiovascular disease, with an increasing prevalence with age, but with easily available medications to control it. Adverse effects of these medications do limit their use, in particular hyponatremia due to thiazide and thiazide-like diuretics. This is more common in the elderly patients due to a combination of inadequate protein intake and impaired urinary dilution capability, made worse by additional thiazide use. Limiting free water intake and increasing protein intake are often not successful resulting in thiazide avoidance. Daily protein supplement is a potential option in this clinical scenario. We describe the protocol for a feasibility study to explore this option.

**Methods:**

This is a single-arm, prospective, open-label proof-of-concept trial, including elderly patients with thiazide diuretic-induced hyponatremia. Forty patients will be enrolled and receive a bottle of a protein supplement daily, providing 120 mmol of solutes and permitting an extra 163 mL free water loss, for 4 weeks. The main outcome measures will be (1) feasibility for enrollment, (2) safety of the intervention, and (3) potential efficacy of the intervention in improving hyponatremia. Secondary outcome measures will include changes in urine osmolality, body weight, and urea measurements.

**Discussion:**

Thiazide diuretic-induced hyponatremia is an important adverse effect, with significant clinical impact, such as delirium and falls, and limits the use of these potent antihypertensive agents. There are little data on the effect or safety of protein supplementation and also on whether a trial of this is feasible. The results of this proof-of-concept feasibility trial will help plan and execute a larger definitive trial to test protein supplementation as an effective strategy in this condition.

**Trial registration:**

The trial is registered with Clinical trials, registration identifier: NCT02614807.

## Background

Hypertension (HTN) is highly prevalent among adults and is the most important modifiable risk factor for cardiovascular events, in particular stroke [1]. The prevalence of HTN increases with age: in those aged 20–24 years it is 0.5%, but it increase to 54% in the elderly (older than 65 years) and further rises to 75% in the very elderly (> 80 years old) [1]. Large randomized controlled trials (RCTs) in the elderly and very elderly have shown clear benefits in vascular outcomes, namely strokes, with pharmacological treatment of HTN [2–5]. These RCTs also established new targets for treatment of blood pressure (BP) in the elderly (< 140/90 mmHg) and very elderly (< 150/90 mmHg). Finally, these RCTs have demonstrated the safety of the use of three BP-lowering drug classes which are now the mainstay of treatment of HTN in these populations [2–5]. These are thiazide and thiazide-like diuretics (TDs, such as hydrochlorothiazide, chlorthalidone, and indapamide), dihydropyridine calcium channel blockers (CCB), and the blockers of the renin-angiotensin system (RAS) [2–5].

However, hyponatremia is a relatively common adverse effect of clinical significance in the treatment of HTN with TDs in the elderly. Hyponatremia refers to low plasma sodium concentration and represents free water excess in the body. It may cause fatigue, nausea, headaches, and muscle weakness if mild, but may lead to serious neurological complications including seizures, coma, and death in its most severe form. Its prevalence increases with age to as high as 20% [6–8]. Given the overall prevalence of HTN among the elderly, this does represent a significant clinical problem. Unfortunately, the other two BP-lowering drug classes with proven efficacy and safety in these patients also have limiting and debilitating adverse effects. Briefly, 1 in 5 patients prescribed with dihydropyridine CCB will develop debilitating ankle edema. RAS blockade also is associated with many side effects ranging from debilitating cough (about 1 in 10), hemodynamically mediated renal impairment, and severe hyperkalemia to rare cases of life-threatening angioedema. In most cases where adverse effects occur, the drugs need to be stopped. Hence, a large proportion of the elderly patients referred to specialized hypertension clinics are labeled as having difficult to treat HTN because of these drug allergies or intolerance.

Hyponatremia induced by TDs in the elderly is multifactorial [6–8]. These patients have age-related decrease in urinary dilution capability (in other words limitation to excrete free water) at baseline, and this is further diminished by the direct effect of the TDs on the sodium chloride co-transporter in the distal convoluted tubule. Practically, this means that these patients require more urinary solute (sodium, potassium, urea) to excrete water. However, dietary solute intake is often quite low among the elderly due to low electrolyte intake and low protein intake on account of decreased appetite as well as due to the low sodium diets that are often prescribed as non-pharmacologic therapy for HTN [6–8]. As a result, elderly patients on TDs may become hyponatremic with as little as 1.5 L per day of water intake. There is a lack of consensus for the right approach to management of hyponatremia in this setting. Increasing salt intake may worsen HTN, and a change in dietary habits (decreasing water intake and increasing protein intake) is difficult to implement for the elderly individual. Thus, in the majority of these patients, these offending drugs are simply discontinued and the patients labeled “intolerant” to TDs.

Overall, TD-induced hyponatremia is a significant clinical problem among elderly hypertensives which prohibits their use in a significant proportion at best and may have serious adverse outcomes if not recognized or treated in a worst case scenario. Knowing the pathophysiology of TD-induced hyponatremia in elderly HTN patients, there is a case for a proof-of-concept clinical trial to test a unique, simple to understand and administer, and relatively affordable approach to rectify this adverse event. Provision of a concentrated dietary supplement which is high in protein should increase free water excretion by the kidneys by providing an increased solute load in the form of urea, the metabolic end product of protein catabolism. This will not change the patient’s diluting capacity, but will allow for a larger volume of free water to be excreted due to the osmotic effect of urea in the collecting duct. This approach should result in the slow correction of TD-induced hyponatremia. Direct ingestion of urea itself has been used and reported to result in an increase of serum sodium levels on the basis of uncontrolled case series [9, 10]. Unfortunately, its bitter taste limits its long-term use, which protein supplements could potentially overcome. There are no prospective trials on the use of protein supplementation nor any data on the possible estimate of benefit of protein supplementation for mild TD-induced hyponatremia. Before embarking on a definitive trial, we describe here the protocol for a proof-of-concept feasibility trial. This trial would establish the feasibility of enrolling participants with this condition, as well as provide pilot estimates about safety and potential efficacy which would be needed to design the larger prospective trial.

## Methods

### Study design and setting

**Fig. 1:**
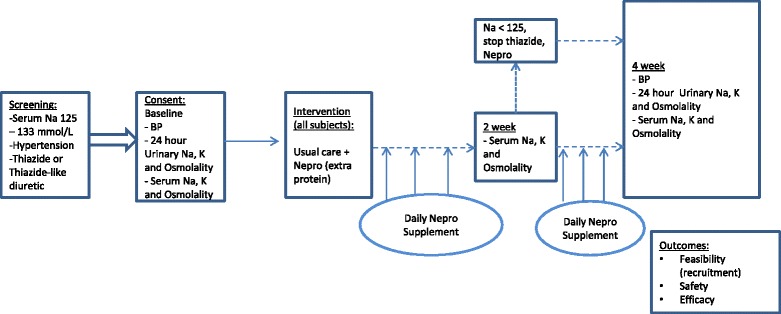
Study flow

The study design is an open-label, single-arm, prospective clinical trial (see Fig. 1). In this proof-of-concept study, we will assess feasibility, safety, and potential efficacy of an intervention of standardized dietary supplement of a bottle (237 mL) of protein supplement (Nepro, Abbott Inc.) a day for treatment of TD-induced hyponatremia in a hypertensive elderly population. A total of 40 consecutive patients with TD-induced hyponatremia over the period of 12–18 months will be recruited for this study from the Renal HTN clinic at the Ottawa Hospital. This clinic has about 1000 patients with hypertension, and TDs are among the most common drug class used. Hyponatremia will be identified by clinic staff and the patients’ primary HTN physician, and if willing to be approached for research, the research staff will be notified.

### Study population

Inclusion criteria:

- Elderly (> 65 years old)
- With diagnosed HTN treated with thiazide (hydrochlorothiazide) or thiazide-like (chlorthalidone and indapamide) diuretic
- Mild to moderate hyponatremia (plasma sodium concentration 125–133 mmol/L)

Exclusion criteria:

- Pregnancy
- eGFR < 45 mL/min/1.75 m^2^
- Other causes of hyponatremia (liver cirrhosis, uncontrolled hypothyroidism, adrenal insufficiency)
- Unable to provide informed consent
- Patients with generalized volume overload who may require immediate changes in diuretic therapy (at the discretion of treating HTN specialist)
- Patients taking drugs which may interfere with urinary sodium excretion (such as carbamazepine, loop diuretics, potassium sparing diuretics, mineralo- and glucocorticosteroids, selective serotonin receptor inhibitors, tricyclic antidepressants, amiodarone, and lithium)
- Patients with moderate to severe hyponatremia (plasma Na concentration < 125 mmol/L) who may require immediate discontinuation of the TD

### Intervention

All recruited patients will receive a standard endorsement of lower fluid intake (< 1.5 L/day) by a hypertension nurse and physician and an additional treatment consisting of a bottle (237 mL) of Nepro a day (supply for 4 weeks will be provided).

### Basic calculations supporting expected potential efficacy of our intervention

In the elderly hypertensive patient (assuming average body weight of 70 kg) with thiazide and thiazide-like diuretic-caused mild to moderate hyponatremia (defined as plasma sodium concentration 125–133 mmol/L), free water excess is around 2.5 L. One bottle of Nepro/day will generate about 120 mosmol to be excreted via urine. This is based on the following calculations: 237-mL bottle of Nepro contains 19 g of protein which will generate 100 mmol of urea, 250 mg of sodium equals to 11 mmol, 250 mg of potassium equals to 6 mmol, and 3 mmol of calcium for a total of 120 mosmol (compare with 250 mosmol with 15 g of urea given every day). Given limited and mostly fixed urinary dilution and concentration between 300 and 800 mosmol/L in the elderly and very elderly, one bottle of Nepro a day will result in an extra 400 mL of urine for a net loss of 163 mL of free water. For a woman weighing 70 kg, with serum sodium of 130 mmol/L, representing approximately 2.75 L of excess free water, the intervention should correct sodium to normal in about 2 weeks. Thus, in theory, over the period of 2 to 4 weeks, the calculated free water excess should be completely eliminated in most patients.

### Outcome measures

There is no prior empiric data about the possible efficacy of this intervention. Hence, we have designed this as a proof-of-concept study. The primary outcomes of the study will be threefold (1) feasibility and (2) safety, in terms of compliance and loss to follow-up and (3) potential efficacy.

- For feasibility, we will examine the number of patients who are eligible and the proportion of those eligible who actually consent for the study.
- For safety, we will examine the proportion of patients who tolerate the Nepro supplement for the duration of the study and who are able to comply with the daily supplementation for the period of study. In addition, we will also report the proportion of patients who might have a worsening of hyponatremia (plasma sodium < 125 mmol/L) either at 2 weeks or at 4 weeks, which will result in immediate discontinuation of the TD.
- For potential efficacy, we will assess the cumulative proportion of participants whose serum sodium either normalizes (> 133 mmol/L) by 4 weeks or who exhibit a decrease in deficit by 50% compared to baseline level.

Secondary outcomes will include changes in urine osmolality, urine sodium, urea, and potassium excretions and plasma potassium, urea, office blood pressure, and body weight.

### Measurements

Plasma sodium, potassium, urea, creatinine, osmolality, and urinary counterparts from 24-h urine collection will be assessed at baseline and at 4 weeks post dietary intervention, which will serve to provide estimates of potential efficacy. Plasma sodium, potassium, and osmolality will also be measured at 2 weeks for safety purposes (to ensure no patient has moderate to severe hyponatremia < 125 mmol/L). If sodium drops to < 125, or patient has any neurological symptoms at any stage, TD will be stopped and patient will be treated according to standard of care (safety outcome). Office blood pressure will be evaluated at same time points.

### Ethical issues and trial registration

The pilot trial will be conducted in accordance with Health Canada’s Good Clinical Practice guidelines, the current Declaration of Helsinki, and the Tri-Council Policy Statement: Ethical Conduct for Research Involving Humans. All patients will be informed that they can withdraw from the study at any time. Patients who agree to participate to the study will provide a written informed consent. The study protocol and informed consent forms have been approved by the Ottawa Health Science Network Research Ethics Board. The trial is registered at the US National Institutes of Health (ClinicalTrials.gov) # NCT02614807.

### Sample size and analytic plan

The study is a proof-of-concept trial, with no prior known efficacy estimates of the intervention. Hence, though no formal sample size estimation is possible, data from this trial will help us to estimate the definitive large trial. The planned sample size will allow us to estimate the rate of efficacy within at most 15%, which we think is necessary to justify a larger trial. The actual observed efficacy will help us plan the definitive trial (if more than 6/40, i.e., > 15%) or decide that the intervention is not significantly efficacious (if < 15%) for further study. A definitive trial in this area will be a randomized controlled trial (RCT) comparing protein supplementation as described in this project with standard of care. From observational registry data, the median rate of change in serum sodium, with medical therapy, while keeping the TD going on, is 1 mEq/L (range 0 to 3 mEq/L) [11]. Thus, assuming a 10% efficacy with the current standard of care and 25% efficacy with the protein supplement, an RCT comparing the two will require a sample size of 226, with 80% power and an alpha of 0.05. The data from the present project with respect to the rate of recruitment will allow a decision on whether this RCT is feasible.

### Progression criteria

We will apply the following specific criteria to make the decision that a definitive trial is worth pursuing, depending on funding. For efficacy, an observed efficacy of greater than 15% (i.e. > 6/40); for safety, the tolerability of the intervention (> 80% are able to tolerate the protein supplement) and serious adverse event (severe or symptomatic hyponatremia requiring immediate discontinuation of TD) rate < 10%. The potential efficacy of > 15% refers to the cumulative proportion of participants whose serum sodium either normalizes (> 133 mmol/L) by 4 weeks or who exhibit a decrease in deficit by 50% compared to baseline level. Lastly, the recruitment rate will help determine the number of sites and length of recruitment period for a definitive trial.

### Study management and patient safety

A trial management group involving the principal investigators (MR, SH), two co-investigators (BM, PM), and a study coordinator will review, implement, and supervise all aspects of this pilot trial. The study coordinator, under the supervision of the principal investigators (SH, MR), will be responsible for receiving, processing, editing, and storing and all the data. All investigational product supplies in the study will be stored in a secure, safe place, under the responsibility of the investigator. The statistical analysis will be conducted by the investigators (SH and TR). The results will be disseminated via conferences and publication in an open access publication and the trial registry; decision for publication lies with the investigators. The reporting of the results will follow the CONSORT statement—extension for Pilot and Feasibility Studies [12].

## Discussion

This is a new approach to identify and test potentially effective method for treatment of TD-induced hyponatremia in elderly hypertensive patients. If successful, this approach will be tested in a large RCT. Positive results could have a major implication for treatment of elderly HTN sparing many of them from being labeled as “TDs intolerant” and deprived of treatment with one of the most established BP-lowering classes. This treatment would be easily available to family physicians and would reduce number of consults referred to HTN Clinics. A negative study outcome also has significant implications for health care delivery and use of resources.

A definitive trial on this topic would compare the protein supplement strategy to the current standard of care (fluid restriction, advice to increase protein intake), with thiazide withdrawal as rescue for severe or intractable cases. Such an RCT would require a sample size of over 200 patients under the assumptions described in the methods, and the current project will demonstrate the feasibility of doing such a definitive RCT.

### Limitations

The study design has certain limitations. Given its pilot feasibility design, it will be inherently underpowered to demonstrate any efficacy; however, the purpose is that the potential efficacy data will allow planning of an appropriately powered trial. Secondly, the intervention might be unsuccessful in increasing sodium levels for a few reasons. Unlike the dose of urea used (15 to 30 g/day corresponding to 250 to 500 mosmol), the protein supplement represents only about 120 mosmol/day extra solute intake. This may result in either an increase without normalizing sodium levels or a longer time to increase sodium levels back to normal. However, there are no human studies evaluating the chronic effect of mild protein loading (leading to extra urea generation) on mild chronic hyponatremia. In contrast to scenarios where urea was used, we will only include patients with mild hyponatremia (125 mmol/L is the lowest threshold for exclusion criteria) and thereby theoretically lower requirement of free water excretion. Additionally, it is possible that participants might concomitantly also increase water intake, which may negate any effect of the intervention, which we will capture by measurement of 24-h urinary volumes (at baseline and end of study). Lastly, any efficacy data based on change from baseline will be susceptible to regression to the mean (given the single-arm study) and will be interpreted with caution.

### Trial status

This trial has received grant funding from the Ottawa Hospital Academic Medical Organization and obtained approval from the institutional review board (the Ottawa Health Sciences Research Ethics Board), and recruitment began in December 2015 and is still ongoing.

## Abbreviations

BP: Blood pressure
CCB: Calcium channel blocker
HTN: Hypertension
RAS: Renin-angiotensin system
RCT: Randomized controlled trials
TD: Thiazide or thiazide-like diuretic
